# Improving district facility readiness: a 12-month evaluation of a data-driven health systems strengthening intervention in rural Rwanda

**DOI:** 10.3402/gha.v8.28365

**Published:** 2015-07-01

**Authors:** Hari S. Iyer, Emmanuel Kamanzi, Jean Claude Mugunga, Karen Finnegan, Alice Uwingabiye, Edward Shyaka, Saleh Niyonzima, Lisa R. Hirschhorn, Peter C. Drobac

**Affiliations:** 1 Partners In Health/Inshuti Mu Buzima, Kigali, Rwanda; 2 Division of Global Health Equity, Brigham and Women's Hospital, Boston, USA; 3 Partners In Health, Boston, USA; 4 Bloomberg School of Public Health, Johns Hopkins University, Baltimore, USA; 5 Ministry of Health, Kigali, Rwanda; 6 Department of Global Health and Social Medicine, Harvard Medical School, Boston, USA; 7 Ariadne Labs, Boston, USA

**Keywords:** global health, health systems strengthening, impact evaluation, resource allocation, Rwanda, rural, sub-Saharan Africa

## Abstract

**Background:**

While health systems strengthening (HSS) interventions are recommended by global health policy experts to improve population health in resource-limited settings, few examples exist of evaluations of HSS interventions conducted at the district level. In 2009, a partnership between Partners In Health (PIH), a non-governmental organization, and the Rwandan Ministry of Health (RMOH) was provided funds to implement and evaluate a district-level HSS intervention in two rural districts of Rwanda.

**Design:**

The partnership provided limited funds to 14 health centers for targeted systems support in 2010; six others received support prior to the intervention (reference). RMOH health systems norms were mapped across the WHO HSS framework, scored from 0 to 10 and incorporated into a rapid survey assessing 11 domains of facility readiness. Stakeholder meetings allowed partnership leaders to review results, set priorities, and allocate resources. Investments included salary support, infrastructure improvements, medical equipment, and social support for patients. We compared facility domain scores from the start of the intervention to 12 months and tested for correlation between change in score and change in funding allocation to assess equity in our approach.

**Results:**

We found significant improvements among intervention facilities from baseline to 12 months across several domains [infrastructure (+4, *p*=0.0001), clinical services (+1.2, *p*=0.03), infection and sanitation control (+0.6, *p*=0.03), medical equipment (+1.0, *p*=0.02), information use (+2, *p*=0.002)]. Composite score across domains improved from 6.2 at baseline to 7.4 at 12 months (*p*=0.002). Across facilities, 50% had composite scores greater than the average score among reference facilities (7.4) at 12 months compared to none at baseline.

**Conclusions:**

Rapid facility surveys, stakeholder engagement, and information feedback can be used for gap analysis and resource allocation. This approach can achieve effective use of limited resources, improve facility readiness, and ensure consistency of facility capacity to provide quality care at the district level.

Global health research and implementation have undergone dramatic changes over the last decade (1). Building on lessons learned from global campaigns to combat AIDS in the early 2000s, policy researchers have called for shifts in thinking from vertical, disease-specific programs to systems-wide interventions to provide effective health services and quality of care (2–4). Reframing global health as a means of achieving equity has allowed research in service delivery to also focus on improving access to care in resource-limited settings through health systems strengthening (HSS) (5, 6).

In order to implement and evaluate HSS interventions, the WHO advocates the use of robust monitoring and evaluation (M&E) systems to track changes across six health systems ‘building blocks’ (WHO BB): health workforce, health service delivery, medicines and medical technology, health information systems, health financing, and leadership and governance (2, 3). Successful implementers have also found that participatory processes involving government and non-governmental stakeholders can improve management of HSS interventions (7–9). While there is agreement that strengthening health systems will lead to better population health outcomes (2, 3), few examples exist that document successful HSS interventions in resource-limited settings (10, 11). Furthermore, little guidance is available to district-level health practitioners seeking to use the results of M&E to improve decision making including resource allocation and equity in health service delivery (12).

In 2005, Partners In Health (PIH), a US-based non-profit organization, partnered with the Rwandan Government (GoR) to strengthen the health system in two rural districts of the country where the health system was relatively weak (13). In 2009, this collaboration was funded by the Doris Duke Charitable Foundation's Population Health Implementation and Training (PHIT) Partnership to implement and evaluate a comprehensive HSS intervention in the two districts. The intervention coupled targeted investments in health systems readiness with interventions targeting quality improvement and data-driven decision making (12–14). The health systems readiness component, evaluated in this paper, aimed to ensure that all facilities in the district health system had what Dr. Paul Farmer has called the ‘staff, stuff, space, and systems’ to provide a comprehensive package of primary health care activities, as defined in this context by Rwanda's Health Sector Strategic Plan (HSSP) (15, 16). Specifically, we hypothesized that rapid assessments of health facility readiness and gap analysis, combined with feedback and engagement with stakeholders resulting in data-driven resource allocation, would lead to improved readiness across all WHO BB and reduce disparities in readiness across health facilities.

## Methods

### Study setting

The HSS intervention was conducted in two rural districts of Rwanda serving an estimated catchment population of 480,000. At the start of the intervention, this population was served by a public health system comprising two district hospitals, 21 health centers, and a network of trained community health workers. Among the health centers, six had received financial and technical support from PIH for at least 1 year prior to the HSS intervention described here (referred to hereafter as ‘reference facilities’). One had started receiving funds just prior to the intervention and so was excluded from analysis. The 14 health centers in the intervention districts that had not previously received HSS support from implementing partners were targeted for the new HSS funds (referred to hereafter as ‘intervention facilities’). At baseline, intervention facilities had an average of 5.4 clinical and 5.7 non-clinical staff, and a total catchment population of 250,000 in 2010 [average: 18,200 (range: 3,920–36,201)].

### Health center strengthening intervention

Finite funds (total $650,000) were available for direct targeted instrumental support to all intervention health centers. Support was provided in the form of investments in specific facility infrastructure and services, guided by the Rwanda HSSP and WHO BB frameworks (Table 1). Support varied by facility and included a range of options within each BB. Health center administration and management support included generator fuel, funds for communication (mobile and Internet), and office supplies. Infrastructure and medical equipment included renovations, repairs, water and power systems, and medical. Human resource support was given in the form of salary support for clinical and non-clinical staff. Medicines included pharmaceuticals and medical equipment.

**Table 1:** Facility survey mapped to WHO building blocks

WHO building block	Facility readiness domain	Data elements	Examples of investment to fill identified gap
Service delivery	Infrastructure	Facility repair, availability of electricity, availability of safe water	Building renovations (such as re-roofing, re-painting, re-flooring), provision of utilities such as power and water supply through purchase of a backup generator, fuel or solar power, construction of rainwater collection containers
Service delivery	Clinical services	Services offered as expected, range of services offered	Trainings/mentorship in clinical areas through MESH program (28, 29)
Health workforce	Human resources	Number of clinical and non-clinical staff, staff retention	Salary support to ensure that health centers had staffing levels more in line with MOH norms
Health information systems, leadership/governance	M&E/information use	Data use, information availability	Provision of salaries for EMR data officers, trainings in monitoring and evaluation and data quality, laptop computers
Service delivery	Laboratory	Availability of gloves, needles, laboratory tests and reagents	Provision of laboratory equipment and reagents
Service delivery	Infection and sanitation control	Availability of hand washing stations, latrine functionality	Build latrines and toilets
Service delivery	Medical equipment	Availability and functionality of 15 essential pieces of equipment, presence of functional equipment	Provision of medical durables, such as hospital beds, neonatal warming lamps, delivery tables, and mosquito nets
Access to essential medicines	Pharmacy	Pharmaceuticals in stock, management of supplies	Provision of technical support, refrigerators, essential medicines, free specialty non-formulary medicines to district pharmacies
Financing	Social services	Support for patients unable to pay, social services	Assistance with the payment of mutuelle (Rwandan national health insurance) and ticket moderateur fees (co-payment for medical visits and treatment) for indigent patients
Service delivery	Referral	Ambulance availability, communication systems	Communications funding, purchase of ambulance to meet goal of five ambulances per district, dedicated nurse to follow referrals to reference hospitals
Leadership/governance	Management	Management practices	Technical assistance to GoR district leadership in implementing district health systems strengthening plan, coordination of regular meetings to review health center's functioning

A short health facility survey was developed from the WHO Services Provision Assessment and the GoR district HSS tool, an annual survey based on national standards for health service delivery (15) to rapidly identify gaps at health centers. Our 77-question survey was mapped to the WHO BB Framework and grouped into one of 11 domains identified as important for health facility readiness: infrastructure, clinical services, laboratory equipment, pharmacy/supply chain, medical equipment, human resources, management, data use/information, infection control/sanitation, social services, and referral systems (Table 1). Forty-nine of the 77 questions corresponded to the 11 domains and were used to calculate scores for each domain. Remaining questions represented priority information for targeted programs, rather than multi-service HSS. Scores were normalized, allowing for a possible score from 0 to 10 (0 meaning no GoR norms were met and 10 that all priority standards were achieved).

Prior to implementation, the tool was translated from English to Kinyarwanda and pilot tested in one intervention area health center. A team of three trained data officers administered the tool through interviews with health center managers and key personnel, direct observation, and review of documents and registers. The survey took roughly 4 h to complete. Data were entered into Access databases and analyses were presented to stakeholders using Excel dashboards.

Stakeholder meetings were held to review data collected using the baseline survey and prioritize resource allocation at district level to determine how much investment each health center received, and at health center level to decide where to target funds. Meetings included the director of the district health unit, medical director of the district hospital, heads of health centers (*titulaires*), and local health officials who represented community interests, with decisions presented to the mayor (the elected executive leader of the district government). These meetings allowed health center staff and leadership to provide input and guide prioritization of the specific support needed to improve patient care at their health centers. Meetings also allowed government officials to update PIH staff about other implementing partners working in the area able to address identified needs, allowing better coordination of the work and funding across all district partners. Decisions made during meetings also included engagement of new partners or government programs to support projects that were beyond the reach of the PHIT HSS funds.

Following resource allocation decisions, implementation of HSS activities was incorporated into the district's annual work plans. For logistical reasons, initiation of facility investments at intervention health centers was phased over a 12-month period (July 2010 through June 2011): six health centers received support in the first quarter of the year, two in the second quarter, four in the third quarter, and the remaining two received support in the final quarter. Each facility received 12 months of support following initiation of investments. The 12-month phased approach allowed time to help decision makers utilize these data for current allocation of MOH and other financial resources, and also to train them to do so in the future. The baseline assessment was completed from October to December 2010 at all health centers delivering care across the two districts. Following the initial gap analysis and completion of resource allocation, the facility survey continued to be used on a quarterly basis as a tool for M&E of the HSS work.

### Evaluation

In order to understand the cost implications of our HSS intervention, we analyzed cost data from the PHIT economic evaluation of the health system in the intervention area (13, 17). Cost surveys were designed to capture existing capital, funding sources (GoR, private, external partners, PIH/PHIT and other sources) and expenditures of health facilities. Notably, our funding data captured in-kind donations by funding source to better estimate health system costs (17). We report on total facility funding for the first and second years of the PHIT partnership (July 2009–June 2010: prior to addition of HSS support, and July 2010–June 2011: including the initial HSS-related support) (13, 17) to compare costs before and after the HSS intervention. Costs were adjusted for health center catchment area to determine cost per capita. We report costs in 2011 USD.

Our primary outcome was the change in the average (composite) facility score at the 14 intervention facilities (calculated by averaging across the 11 domains of facility readiness identified in our survey) from baseline to 12 months. Our secondary outcomes were the change in individual domain scores over time; additionally, we tested for correlations between change in score and change in direct funds received by health centers over the study period. In order to determine if improvements were sustained beyond the 12-month evaluation, we also calculated facility readiness scores for intervention facilities at 24 months and 36 months. We also compared the facility scores of intervention facilities at 12 months to the facility scores among the reference health centers at baseline to determine if our intervention succeeded in bringing the intervention facilities to similar levels of readiness as those of reference facilities which had received support from PIH prior to the intervention.

We used Wilcoxon signed rank tests to assess changes in intervention facility readiness scores from baseline to 12 months and the Pearson correlation coefficients with Fisher's z transformation to test for correlations between changes in facility readiness scores and changes in health center funding over a 12-month period. We report the correlation coefficient and the 95% confidence interval for the estimates. Statistical analysis was performed using SAS v. 9.2.

## Results

### Baseline scores

Pre-intervention, the composite score across all facilities was 6.2 at baseline for intervention facilities (range: 4.2–7.3) compared to 7.4 (range: 6.2–8.5) for the six reference health centers (*p*=0.03). The facility survey found challenges across critical domains among the 14 intervention health centers (Table 2). Areas of need included infrastructure (2.9 for intervention facilities and 6.7 for reference facilities), social services (2.1 for intervention and 3.3 for reference facilities) and referral systems (5.0 for intervention facilities and 8.3 for reference facilities).

**Table 2:** Change in domain scores from baseline to 12 months among intervention facilities compared to prior supported facilities

WHO building blocks	Facility domain	Reference facilities	Intervention facilities				

		*N*=6	*N*=14				

		Baseline score	Baseline score	Endline score	Difference^a^	*p*^b^
Service delivery	Infrastructure	6.7	2.9	7.0	4.0	**0.001**
	Clinical services	8.6	8.7	9.9	1.2	**0.03**
	Lab services	8.1	7.1	7.6	0.5	0.58
	Infection and sanitation control	7.8	8.1	8.7	0.6	**0.03**
	Medical equipment	7.8	7.5	8.5	1.0	**0.02**
	Social services	3.3	2.1	4.6	2.5	0.20
	Referral	8.3	5.0	4.3	−0.7	0.73
	Management	6.7	7.9	8.3	0.5	0.63
Health workforce	Human resources	8.9	5.0	6.1	1.1	0.51
Health information systems	M&E/information use	7.6	6.3	8.4	2.0	**0.002**
Access to essential medicines	Pharmacy	7.1	7.9	8.4	0.5	0.11
Composite score (all domains)	Total	7.4	6.2	7.4	1.2	**0.002**

a Numbers may not add up due to rounding

b Wilcoxon signed rank test for non-parametric paired samples.

Bold text refers to *p*-value<0.05.

### Resource allocation

**Fig. 1:**
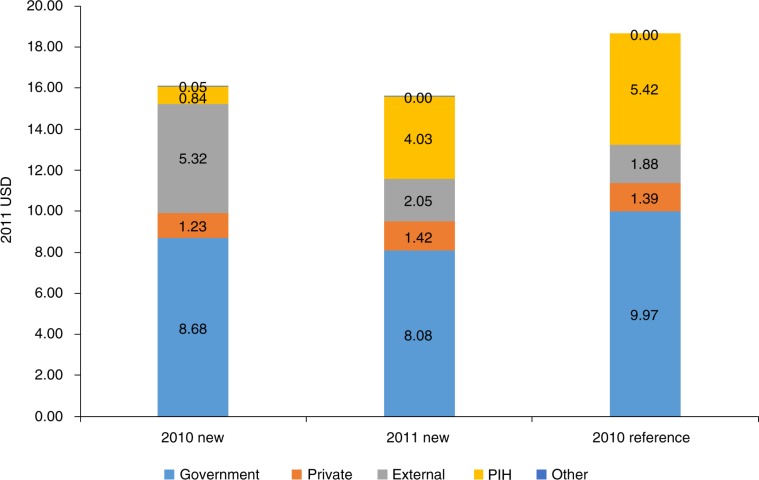
Per capita HS investments* in 2011 USD by funding source among intervention facilities (*N*=14) over a 12-month period to reference facilities (*N*=6) at baseline. *Includes in-kind donations.

During the 12-month intervention period, the partnership's investment in health center readiness was $4.03 per capita, though allocation varied across the 14 health center catchments (median: $4.01, range: $1.00–$15.96 per capita). This comprised 26% of total per capita health funding ($15.58) in the intervention catchment area. Per capita government funding remained relatively constant from the pre-intervention year ($8.68, or 54% of total) to the intervention year ($8.08, 52%), while external funding declined from $5.32 (33%) per capita to $2.05 (13%). The latter reflected a one-time, in-kind contribution of medical equipment and furniture by a multilateral partner during the pre-intervention year that was received by most health centers in the country. As such, despite the new investment from the HSS intervention, total per capita funding in the catchment area declined 3% from the pre-intervention year to the intervention year ($16.12–$15.58). Total per capita funding in the intervention catchment was 17% less than that of the reference facility area, though distribution of funding sources was similar (Fig. 1).

### Change in scores

**Fig. 2:**
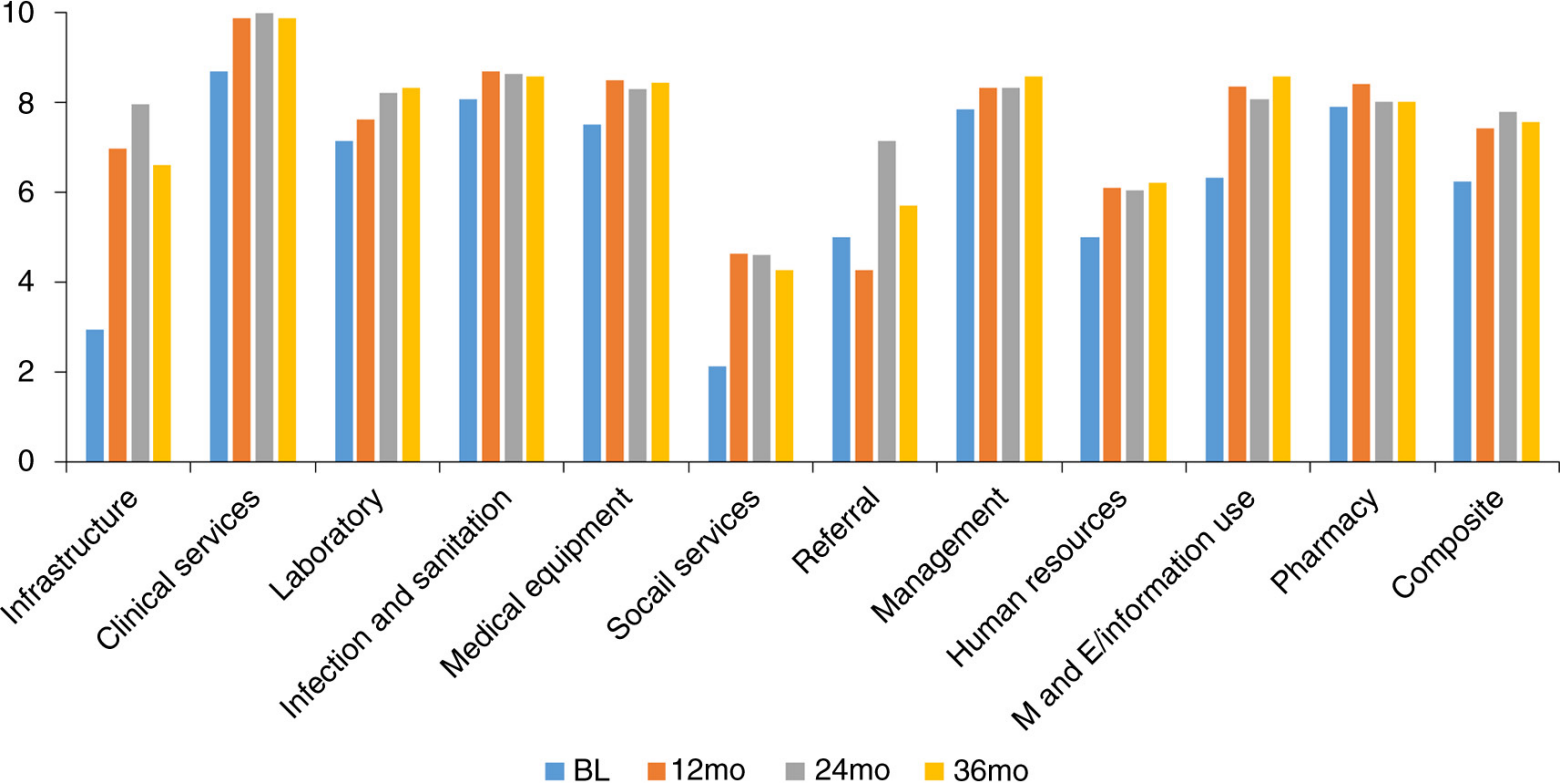
Sustainability in Facility Readiness Improvement among Intervention Facilities (*N*=14) after 36 months.

Despite a slight decline in overall health center funding, composite scores increased from baseline to 12 months at the 14 intervention health centers (difference = 1.2, *p*=0.002). Multiple domain scores also showed significant improvement in the intervention facilities (infrastructure: 4.0, *p*=0.001; clinical services: 1.2, *p*=0.03; infection and sanitation control: 0.6, *p*=0.03; medical equipment: 1.0, *p*=0.02; M&E: 2.0, *p*=0.002) (Table 2). Across the 14 intervention health centers, 50% had composite scores greater than the average score among reference facilities (7.4) at 12 months; none of the intervention health centers had a higher composite score at baseline. Improvements remained relatively stable at 24 months and 36 months (Fig. 2). At 24 months, the composite score for intervention facilities increased to 7.8 and at 36 months, the composite score was 7.6.

### Factors correlated with change in composite facility score

We assessed the correlation between change in composite score, change in direct funding, and change in each individual domain score to determine whether any of these factors was significantly associated (Table 3). We found a trend towards positive correlation between change in score and change in PHIT funding (*r*=0.40, 95% CI: [−0.17, 0.77]), but this was not statistically significant. Baseline score was negatively correlated with change in score (*r*=− 0.71, 95% CI: [−0.90, −0.28]). Changes in several specific domains were also significantly correlated with changes in composite score (HR: *r*=0.52, 95% CI: [−0.02, 0.82], clinical services: *r*=0.59, 95% CI: [0.09, 0.86], social services: *r*=0.68, 95% CI: [0.23, 0.89]).

**Table 3:** Correlation between difference in composite facility score over 12 months and other covariates among 14 intervention facilities

	*r*	95% CI	*p*^a^
Difference in composite facility score			
**Baseline score**	−**0.71**	−**0.90**, −**0.28**	**0.003**
Baseline PHIT HSS direct funding	−0.16	−0.64, 0.41	0.58
Change in PHIT HSS direct funding	0.40	−0.16, 0.77	0.14
Change in equipment score	0.46	−0.09, 0.80	0.09
**Change in HR score**	**0.52**	−**0.02, 0.82**	**0.049**
Change in infrastructure score	0.14	−0.42, 0.63	0.62
Change in M&E/information score	−0.17	−0.65, 0.39	0.54
**Change in laboratory score**	**0.51**	−**0.03, 0.82**	**0.054**
Change in management score	0.33	−0.24, 0.73	0.24
Change in pharmacy score	0.19	−0.37, 0.66	0.50
Change in referral score	0.46	−0.09, 0.80	0.09
Change in sanitation score	0.06	−0.48, 0.57	0.83
**Change in clinical services score**	**0.59**	**0.09, 0.86**	**0.02**
**Change in social services score**	**0.68**	**0.23, 0.89**	**0.005**

a Bold text denotes *p*-value<0.05.

### Relationship between change in direct funding and change in composite facility score

**Fig. 3:**
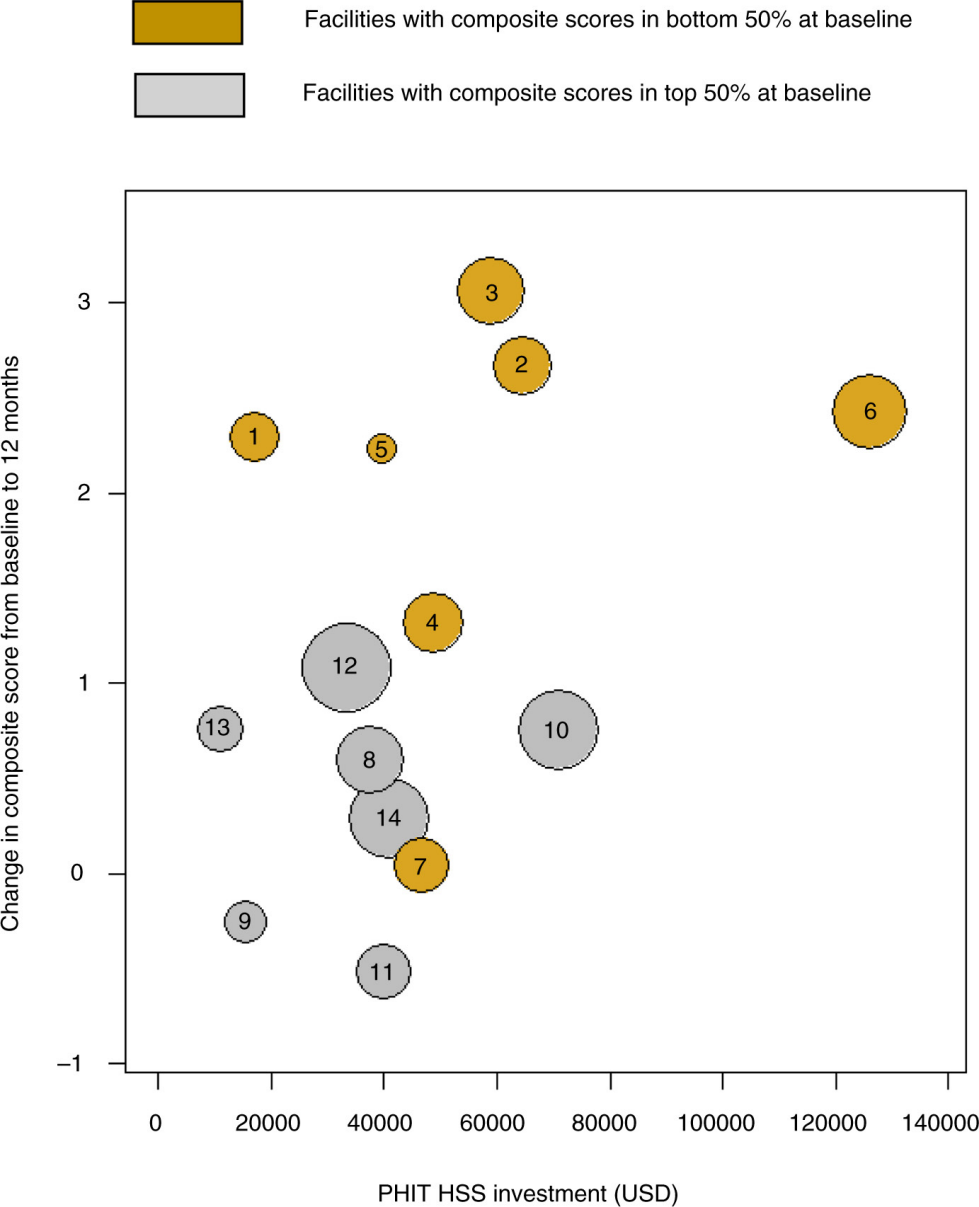
Correlation between change in composite facility score and change in direct PHIT HSS funding among intervention facilities (*N*=14). Number in cells is the rank based on baseline composite score (lowest to highest).

To determine how successful our data-driven decision making strategy was in allocating resources to health centers most in need, we plotted health center change in composite score against change in PHIT HSS funding. We found that the six health centers with the lowest composite scores at baseline were the same six that had the greatest increase in PHIT HSS funding and associated change in composite score at 12 months (Fig. 3).

## Discussion

**Fig. 4:**
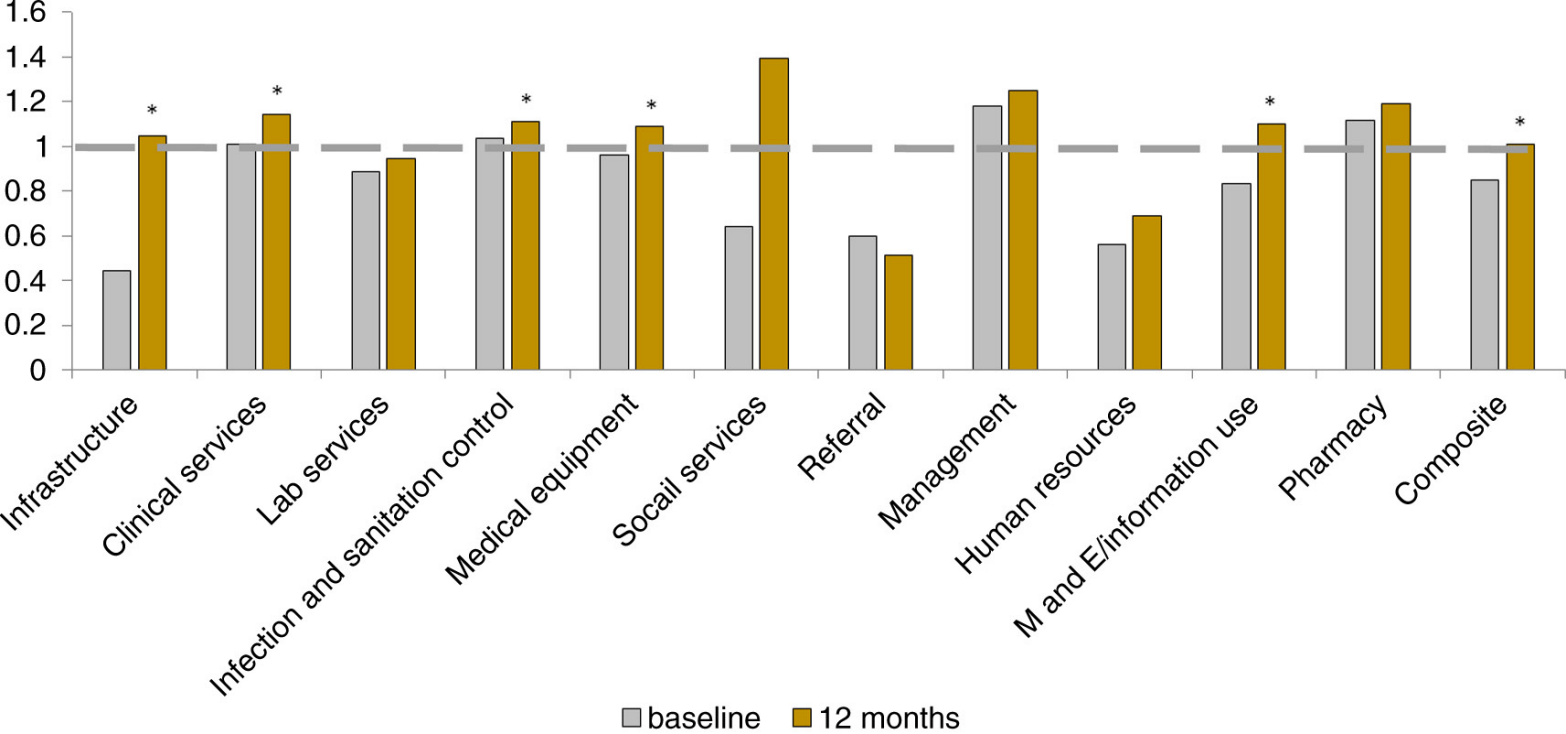
Standardized comparisons of intervention facilities (*N*=14) at baseline and 12 months to baseline facility scores for reference facilities.

We found that implementation of a tool to rapidly assess service availability and facility readiness, combined with engagement of local leaders to utilize these data to identify priority areas for resource allocation, was effective in improving facility readiness in rural Rwanda. Composite scores for intervention facilities had caught up to the baseline scores of the reference facilities, reflective of gains in the infrastructure, medical equipment, social services, and M&E domains (Fig. 4). Our results suggest that we were successful in engaging local leaders to improve equity in health service delivery readiness across the district focusing on health centers targeted for this year-long intervention. Indeed, improvements were greatest among facilities with the lowest readiness scores at baseline (Fig. 3).

Scores among multiple facility domains improved following the health center strengthening in intervention facilities, particularly the infrastructure, clinical services, M&E, medical equipment and infection and sanitation domains. These were all identified as priority areas during the quarterly stakeholder meetings, and were able to be addressed by our implementation teams. Infrastructure was repeatedly listed as a high priority area for improvement as many health centers were in need of repairs or had too little space to accommodate their patients. Certain areas, like social support and human resources, were not amenable to a one-off investment and required sustained follow-up, which could have led decision makers to favor other areas of investment. We found that referral scores and social support scores had less improvement over the course of the intervention. These findings suggest that these among all of the areas of need, referral networks and social support were not prioritized by the partnership for improvement in this intervention. In addition, referral networks may require a broader district-level approach given the need to engage all three levels of care delivery (community, health center, and district hospital).

Facility improvements were achieved through relatively low financial investments, never amounting to more than a third of overall health systems funding in the intervention area. Interestingly, overall per capita spending in the intervention area declined slightly in the intervention period, relative to the prior year. This was due to a one-time, large in-kind donation of furniture and equipment by an external partner in the pre-intervention year. Indeed, government funding remained relatively constant, and has increased in the years since. That the 14 intervention health facilities achieved significant increases in readiness scores despite a decline in overall funding suggests that our HSS intervention may have created more value than prior investments (18). The intervention's strategy of targeting investment to measured health system gaps using a participatory process facilitated smarter resource allocation.

While the facility survey tracked the improvement in facility readiness, our intervention also introduced many other positive changes to government district health system management that persisted beyond the financial investments made to intervention facilities. For the first time, many *titulaires* were introduced to a data-driven method of resource allocation and advocacy. The facility survey, which was originally introduced for gap analysis, has now been incorporated into routine M&E by district teams. Gains in M&E/information use and management facility domain scores have increased at 24 and 36 months, highlighting the sustained improvements that have resulted from this intervention (Fig. 2). The quarterly meetings have strengthened the relationship between PIH and the district GoR leadership, and have created an environment amenable to greater data use for decision making.

Our study also demonstrates the power of the facility survey to highlight disparities in facility readiness across a large geographic area. When linked to a commitment to stakeholder engagement and shared decision making, the survey empowers health systems managers to respond to these gaps in an equitable manner through targeted resource allocation. While others have studied the breakdown of HSS spending using the WHO BB Framework (19, 20) and described challenges in attributing funding to health outcomes (21), these studies have focused on national level health financing rather than district level. Our findings demonstrated that in our districts, we were successful in targeting low performing health centers as those with low baseline scores had the greatest changes in funding. We attribute this to involving government partners during the entire process and our use of facility scores as an evidence-based advocacy tool.

Other researchers in Uganda have reported on how improvements in health worker skills gained through engagement with data are correlated with self-efficacy and motivation, and that creating a culture of data use were correlated with high worker motivation (22). For us, facility scores provided an objective metric that could be used to determine which health centers were most in need of support and in what areas, and through our collaborations with our government partners at the district and the leaders of the health centers, we were able to ensure that we were delivering our limited support to those most in need of it. Stronger relationships with our government partners also helped us plan together to transition some of the costs of these investments to the MOH in future years. These improvements in data use and engaging stakeholders to make informed decisions about resource allocation increase the potential for a sustainable impact of our intervention beyond the initial financial investments.

Our study has several limitations. We developed a survey informed by, but not identical to those used by the WHO and others (23–25), meaning that our facility scores may not be generalizable to all contexts (19, 26, 27). This was done because few formal, validated facility surveys that could be done relatively rapidly and at low cost existed at the time of our intervention. We also adapted the survey to reflect Rwanda's national standards and the district management tool, which was under development at the time. The domain scores measure very narrow aspects of each domain in order to serve their purpose – providing decision makers with information about underperforming areas of facility readiness. Tracking in-kind donations and sources of financing at health centers was challenging due to poor record keeping, meaning that we may have underestimated funding at some health centers. Our sample size is small – we only present data for 20 health centers in two low-income, rural districts of Rwanda, limiting generalizability to other contexts. We did not report on changes in facility readiness among reference facilities over the same time period for two reasons: 1) reference facilities had already achieved a high level of readiness through intensive support prior to the intervention (Table 1), and 2) the investments made at reference facilities were much higher than at intervention facilities (Fig. 1). Instead, we chose to use the level of readiness achieved by reference facilities as a standard against which to measure progress towards improved facility readiness among intervention facilities. With a lack of a true control group, we are cautious in our interpretation of these results. Finally, we make no mention here of effects of the health center strengthening on quality, resource utilization by patients, or health outcomes.

In summary, we describe the results of a data-driven method for district-level HSS. We show that components of the WHO health service delivery and health information building blocks are amenable to improvement through a 1-year, intense targeted allocation of modest funds. We demonstrate that through government and non-profit stakeholder engagement and feedback, it is possible to ensure that the funds are distributed in an equitable fashion, allowing the worst performers to catch up with the best performers and that priority areas are targeted. Further studies are planned to assess the impact of this intervention on patient care and ultimately, population health outcomes.

## References

[1] Farmer P, Kleinman A, Kim JY, Basilico M (2013). Reimagining global health: an introduction. Vol. 26.

[2] World Health Organization (2007). Everybody's business: strengthening health systems to improve health outcomes. WHO's framework for action.

[3] de Savigny D, Adam T (2009). Chapter 1, Systems thinking for health systems strengthening: An Introduction. Systems thinking for health systems strengthening.

[4] Travis P, Bennett S, Haines A, Pang T, Bhutta Z, Hyder A (2004). Overcoming health-systems constraints to achieve the Millennium Development Goals. Lancet.

[5] Gwatkin DR, Bhuiya A, Victora CG (2004). Making health systems more equitable. Lancet.

[6] 
Swanson RC, Bongiovanni A, Bradley E, Murugan V, Sundewall J, Betigeri A (2010). Toward a consensus on guiding principles for health systems strengthening. PLoS Med.

[7] Doherty T, Chopra M, Nsibande D, Mngoma D (2009). Improving coverage of the PMTCT programme through a participatory quality improvement intervention in South Africa. BMC Public Health.

[8] Youngleson MS, Nkurunziza P, Jennings K, Arendse J, Mate KS, Barker P (2010). Improving a mother to child HIV transmission programme through health system redesign: quality improvement, protocol adjustment and resource addition. PLoS One.

[9] Scott V, Chopra M, Azevedo V, Caldwell J, Naidoo P, Smuts B (2010). Scaling up integration: development and results of a participatory assessment of HIV/TB services, South Africa. Health Res Policy Syst.

[10] Bennett S, Agyepong IA, Sheikh K, Hanson K, Ssengooba F, Gilson L (2011). Building the field of health policy and systems research: an agenda for action. PLoS Med.

[11] Bennett S, Adam T, Zarowsky C, Tangcharoensathien V, Ranson K, Evans T (2008). From Mexico to Mali: progress in health policy and health systems research. Lancet.

[12] Bassett MT, Gallin EK, Adedokun L, Toner C (2013). From the ground up: strengthening health systems at district level. BMC Health Serv Res.

[13] Drobac PC, Basinga P, Condo J, Farmer PE, Finnegan KE, Hamon JK (2013). Comprehensive and integrated district health systems strengthening: the Rwanda Population Health Implementation and Training (PHIT) Partnership. BMC Health Serv Res.

[14] Binagwaho A, Nutt CT, Uwaliraye P, Wagner CM, Nyemazi JP (2013). Taking health systems research to the district level: a new approach to accelerate progress in global health. BMC Health Serv Res.

[15] Government of Rwanda Ministry of Health (2009). Health Sector Strategic Plan (HSSP II), July 2009–June 2012.

[16] Farmer P (2015). Who lives and who dies. Lond Rev Books.

[17] Lu C, Tsai S, Ruhumuriza J, Umugiraneza G, Kandamutsa S, Salvatore PP (2014). Tracking rural health facility financial data in resource-limited settings: a case study from Rwanda. PLoS Med.

[18] Kim JY, Farmer P, Porter ME (2013). Redefining global health-care delivery. Lancet.

[19] Shakarishvili G, Lansang MA, Mitta V, Bornemisza O, Blakley M, Kley N (2011). Health systems strengthening: a common classification and framework for investment analysis. Health Policy Plan.

[20] Warren AE, Wyss K, Shakarishvili G, Atun R, de Savigny D (2013). Global health initiative investments and health systems strengthening: a content analysis of global fund investments. Global Health.

[21] Ataya N, Aluttis C, Flahault A, Atun R, Haines A (2014). Improving the assessment and attribution of effects of development assistance for health. Lancet.

[22] Hotchkiss DR, Aqil A, Lippeveld T, Mukooyo E (2010). Evaluation of the Performance of Routine Information System Management (PRISM) framework: evidence from Uganda. BMC Health Serv Res.

[23] Nickerson JW, Adams O, Attaran A, Hatcher-Roberts J, Tugwell P (2015). Monitoring the ability to deliver care in low- and middle-income countries: a systematic review of health facility assessment tools. Health Policy Plan.

[24] Mutale W, Stringer J, Chintu N, Chilengi R, Mwanamwenge MT, Kasese N (2010). Application of balanced scorecard in the evaluation of a complex health system intervention: 12 months post intervention findings from the BHOMA intervention: a cluster randomised trial in Zambia. PLoS One.

[25] Loveday M, Padayatchi N, Wallengren K, Roberts J, Brust JC, Ngozo J (2014). Association between health systems performance and treatment outcomes in patients co-infected with MDR-TB and HIV in KwaZulu-Natal, South Africa: implications for TB programmes. PLoS One.

[26] O'Neill K, Takane M, Sheffel A, Abou-Zahr C, Boerma T (2013). Monitoring service delivery for universal health coverage: the Service Availability and Readiness Assessment. Bull World Health Organ.

[27] Mounier-Jack S, Griffiths UK, Closser S, Burchett H, Marchal B (2014). Measuring the health systems impact of disease control programmes: a critical reflection on the WHO building blocks framework. BMC Public Health.

[28] Magge H, Anatole M, Cyamatare FR, Mezzacappa C, Nkikabahizi F, Niyonzima S (2015). Mentoring and quality improvement strengthen integrated management of childhood illness implementation in rural Rwanda. Arch Dis Childhood.

[29] Anatole M, Magge H, Redditt V, Karamaga A, Niyonzima S, Drobac P (2013). Nurse mentorship to improve the quality of health care delivery in rural Rwanda. Nurs Outlook.

